# Timely Diagnosis of Pneumoperitoneum by Point-of-care Ultrasound in the Emergency Department: A Case Series

**DOI:** 10.5811/cpcem.2021.4.52139

**Published:** 2021-07-27

**Authors:** Jung Yum, Taryn Hoffman, Leily Naraghi

**Affiliations:** Maimonides Medical Center, Department of Emergency Medicine, Brooklyn, New York

**Keywords:** Emergency medicine, point of care ultrasound, pneumoperitoneum, free intraperitoneal air, enhanced peritoneal stripe sign, case series

## Abstract

**Introduction:**

Pneumoperitoneum is a life-threatening diagnosis that requires timely diagnosis and action. We present a case series of patients with perforated hollow viscus who were accurately diagnosed by emergency physicians using point-of-care ultrasound (POCUS) while in the emergency department (ED).

**Case Series:**

Three elderly patients presented to the ED with the complaints of syncope, abdominal pain with constipation, and unresponsiveness. The emergency physicians used POCUS to diagnose and then expedite the necessary treatment.

**Conclusion:**

Point-of-care ultrasound can be used by emergency physicians to diagnose pneumoperitoneum in the ED.

## INTRODUCTION

Perforation of a hollow organ such as stomach or intestine is an acutely life-threatening condition. It requires urgent surgical intervention in up to 90% of patients and carries a high rate of associated peritonitis, sepsis, and death.^1^ Despite advances in diagnostic and surgical technologies, morbidity and mortality associated with perforated viscus remains as high as 20–36%.^2^ As a result, rapid diagnosis and early management is critical for positive patient outcomes.

The diagnosis of pneumoperitoneum has traditionally been made by upright chest radiograph (CXR) and/or computed tomography (CT). However, there are many factors in the emergency department (ED) that make it difficult to get these studies done in a timely manner. Ultrasound is more readily accessible and can be used to rapidly diagnose pneumoperitoneum and expedite management in the ED.

Sonographic diagnosis of pneumoperitoneum is made by first scanning the patient in supine position in both the right upper quadrant and midline of the patient’s abdomen. These areas are ideal as there is the least amount of bowel, especially in the right upper quadrant view between the anterior abdominal wall and liver. Next, the patient is scanned in the left lateral decubitus position for better visualization as air will collect in the least dependent area, the right upper quadrant.^3^

There are three main sonographic signs that indicate the presence of pneumoperitoneum. The first is referred to as the enhanced peritoneal stripe sign (EPSS).^1^ As free air rises anteriorly in a supine patient, it makes contact with the peritoneal lining, creating a characteristic thick hyperechoic line.^1^,^3^,^4^ Second is the shifting phenomenon. As the patient moves from one position to another, air will rise to the least dependent position. This results in shifting of the EPSS with movement, most notably from the anterior abdomen to the lateral aspect of the liver when the patient moves from supine to left lateral decubitus position.^4^ The final sign is the ring-down artifact. In some cases, the presence of air can cause echoes at the air-to-soft tissue interface, causing posterior reverberation artifacts.^3^,^4^

Most studies evaluating the role of ultrasound in diagnosing pneumoperitoneum have been performed by radiologists. There is limited data available to demonstrate the role of point-of-care ultrasound (POCUS) in diagnosing pneumoperitoneum in the ED.^4^ In this case series, we present three patients who had pneumoperitoneum diagnosed by an emergency physician using POCUS, which helped expedite further care in the ED.

## CASE SERIES

### Case 1

A 79-year-old male presented to the ED with a chief complaint of syncope. He had a past medical/surgical history of bladder cancer with prior neoadjuvant chemotherapy and subsequent consolidative cystoprostatectomy with ileal conduit and left nephrectomy. Prior to the syncopal episode, the patient had been complaining of abdominal pain associated with nausea and vomiting for two days. He was afebrile (98.6ºF) with a heart rate of 135 beats per minute, blood pressure of 85/60 millimeters mercury (mm Hg), respiratory rate of 36 breaths per minute, and oxygen saturation of 94% on nasal cannula. On examination, the patient had an ileal conduit without active signs of infection; however, there was severe diffuse abdominal tenderness to palpation, which raised concern for peritonitis.

**Image 1:**
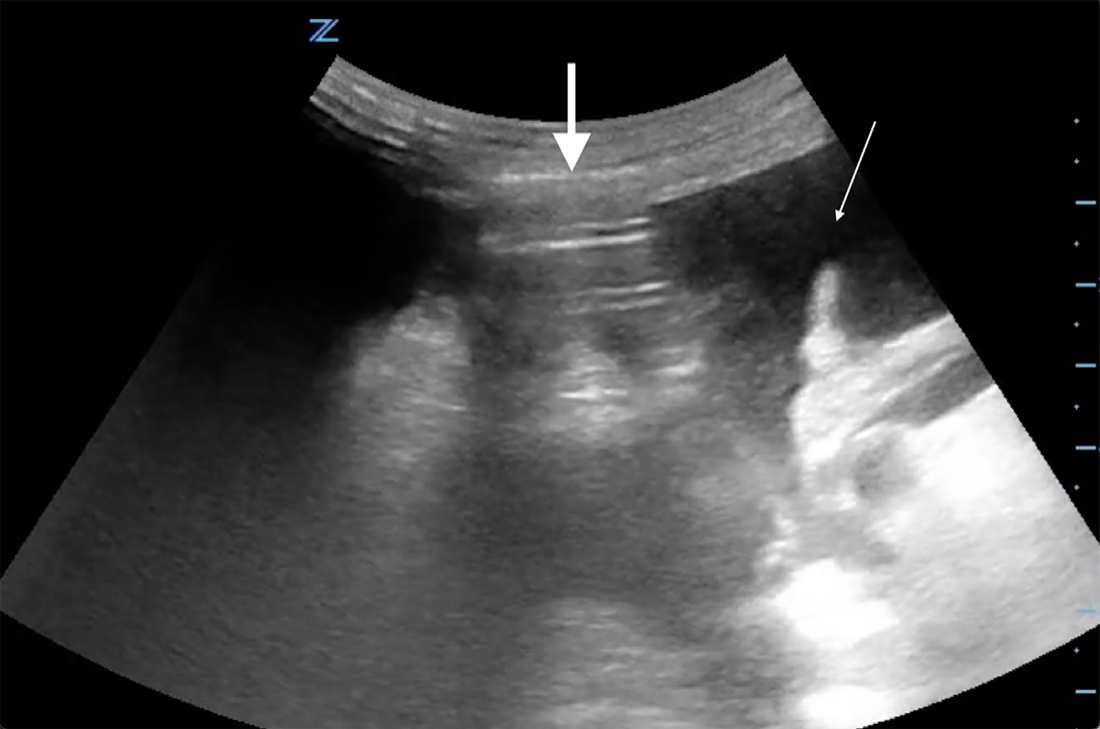
Abdominal ultrasound from Case 1 showing free intraperitoneal fluid (thin arrow) as well as free intraperitoneal air (thick arrow) as demonstrated by the enhanced peritoneal stripe sign and reverberation artifact.

Point-of-care ultrasound showed a positive FAST exam (focused assessment with sonography for trauma) with free fluid in the abdomen (Image 1). It also showed a thick peritoneal stripe with posterior reverberation artifacts consistent with pneumoperitoneum and perforated viscus. Computed tomography of the abdomen/ pelvis was expedited, which confirmed the diagnosis of pneumoperitoneum with viscus perforation. Meanwhile, the patient was given intravenous (IV) fluids, broad spectrum antibiotics, and pain control, and was started on vasopressors. Urology and general surgery were consulted immediately, and the patient was taken to the operating room (OR) emergently for exploratory laparotomy.

**CPC-EM Capsule**

What do we already know about this clinical entity?

*Pneumoperitoneum caused by perforation of hollow viscus is a life-threatening diagnosis, requiring rapid diagnosis and surgical evaluation.*

What makes this presentation of disease reportable?

*We present a case series in which point-of-care ultrasound (POCUS) was used to expedite diagnosis and management in three patients who presented to the emergency department (ED) with hollow viscus perforation and free intraperitoneal air.*

What is the major learning point?

*When concerned about significant pathology such as hollow viscus perforation, ED providers should consider reaching for POCUS during their initial workup of the patient.*

How might this improve emergency medicine practice?

*Understanding and utilizing POCUS during workup can help providers quickly make a diagnosis, thus expediting the definitive management the patient receives and improving their overall care.*

### Case 2

An 80-year-old male was brought into the ED from a nursing home for apparent abdominal pain and two weeks without having a bowel movement. The patient had a medical history of diabetes, hypertension, and dementia. He was unable to provide history due to his dementia. On exam, he was afebrile (97.3ºF) with a blood pressure of 110/70 mm Hg, heart rate of 112 beats per minute and respiratory rate of 24 breaths per minute. His abdomen was nondistended, but it was diffusely tender with voluntary guarding.

**Image 2:**
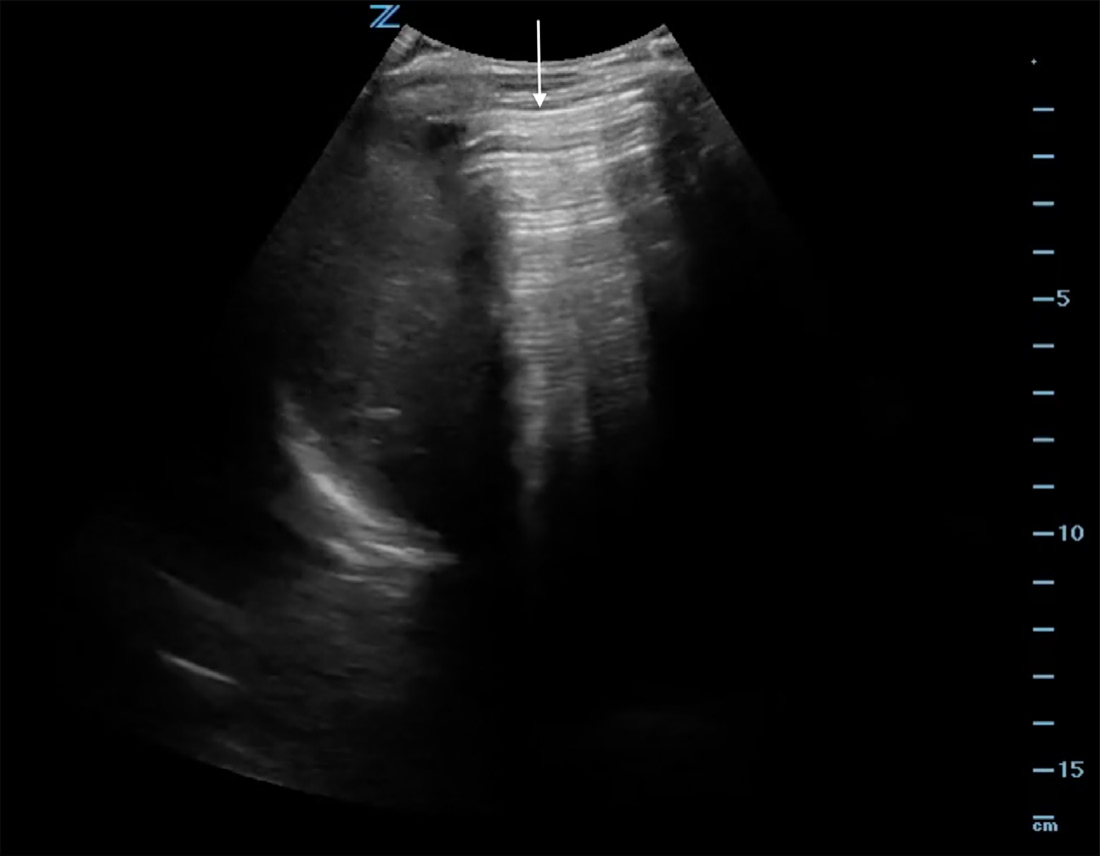
Ultrasound image from Case 2 showing enhanced peritoneal stripe sign in the right upper quadrant, indicative of free intraperitoneal air (arrow).

**Image 3:**
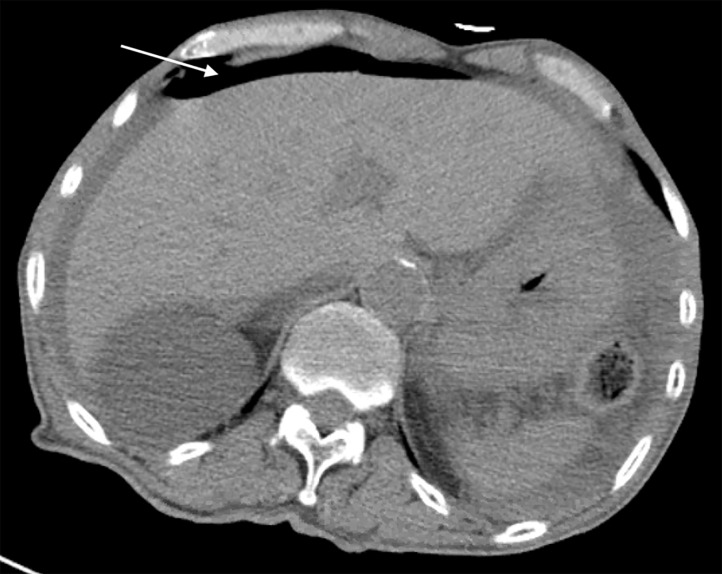
Computed tomography image from Case 2 depicting free intraperitoneal air (arrow).

Point-of-care ultrasound showed a positive FAST with free fluid in the abdomen. It also showed an EPSS suspicious for a perforation (Image 2). He was given IV fluids, analgesia, and broad-spectrum antibiotics. Computed tomography of the abdomen/pelvis confirmed free air and free fluid in the abdomen associated with ischemic jejunum with pneumatosis intestinalis (Image 3). Surgery was consulted, and the patient was taken to the OR for emergent exploratory laparotomy.

### Case 3

An 88-year-old female was brought into the ED after she was found to be unresponsive at home. She had a medical history of hypertension and diabetes. Family reported that the patient had been lethargic for two days associated with poor oral intake. She also had an episode of nausea and bloody vomiting earlier that day. Upon arrival, she was hypoxic to 82% with respiratory rate of 30 breaths per minute even with non-rebreather mask. Heart rate was 88 beats per minute. She was also hypotensive (74/39 mm Hg) with a mean arterial pressure of 50 mm Hg (reference range: 70–100 mm Hg). She was emergently intubated and was started on IV fluids, antibiotics, and vasopressors. Initial abdominal exam was limited due to her mental status, but her abdomen was noted to be distended.

Ultrasound showed EPSS with free air. The patient was started on broad spectrum antibiotics, and the expedited abdominal CT showed large volume pneumoperitoneum with perforation at the distal stomach. Surgery was consulted emergently; after further discussion her family decided against any surgical intervention and requested comfort care in the ED. The patient was subsequently admitted to the hospital for end-of-life care.

## DISCUSSION

Our case series reports the benefits of point-of-care sonographic detection of pneumoperitoneum in the ED. The diagnosis of pneumoperitoneum is usually identified by radiograph and/or CT. On radiographs, pneumoperitoneum is identified by the presence of subdiaphragmatic free air either in the upright or lateral decubitus position. It has been reported that plain radiographs can identify free air as little as one milliliter (mL) of intraperitoneal gas. However, the sensitivity is only 30–59%. Radiography is a reliable diagnostic test only when there are large volumes of free air (sensitivity approaches 100% when there is large volume pneumoperitoneum). In contrast, the sensitivity of CT in detecting free air is 96–100%; In addition, CT is able to identify the specific sites of perforation in 80–90% of cases.^2^

Nonetheless, there are circumstances that delay these imaging modalities significantly. Examples include patient’s inability to stand or sit upright for an upright CXR, limited staffing/transport for CT, and hospital protocols that prioritize CT scanners for time-critical illnesses (eg, code strokes). As seen in two of our cases, there are also instances in which patients are obtunded or unresponsive, making physical exam less reliable even though they may, in fact, have significant underlying pathology. In these three cases, POCUS proved to be an excellent diagnostic tool to use at bedside. Studies have reported that as little as 1–2 mL of intraperitoneal free air can be detected by ultrasound.^3^,^5^ Investigators have also reported a sensitivity of 93% and specificity of 64% for sonographic diagnosis of pneumoperitoneum and an even higher accuracy (sensitivity of 100% and specificity of 99%) when EPSS is present.^2^

While ultrasound is an accessible, cost-effective, and safe diagnostic tool, it comes with its own limitations. It is highly operator dependent with many factors that can prevent good quality images, including rib shadowing, bowel gas, obesity, and subcutaneous emphysema.^3^,^5^ In addition, some critically ill or agitated patients may not tolerate the ultrasound probe on various regions of the abdomens.^3^ Finally, ultrasound cannot identify the exact location of perforation.^2^

This case series highlights the benefits of using POCUS for the timely diagnosis of pneumoperitoneum in the ED to expedite management.

## CONCLUSION

In this case series, POCUS was used to identify signs of pneumoperitoneum, which helped expedite the CT and appropriate treatments and expert consultation in the ED. Thus, POCUS can be used as an extension to the physical examination for earlier detection and management of perforated bowel, especially in patients with concerning or limited abdominal exams. Further studies are required to investigate the effect of performing POCUS on the time to CT and definitive management in patients with abdominal emergencies.

The authors attest that their institution requires neither Institutional Review Board approval, nor patient consent for publication of this case report. Documentation on file.
